# Rational design of novel *Plasmodium falciparum* glutamyl-tRNA synthetase inhibitors for the development of next-generation antimalarial drugs

**DOI:** 10.1371/journal.pone.0334429

**Published:** 2025-12-04

**Authors:** Wesam Nofal

**Affiliations:** Department of Medical Laboratory Technology, Faculty of Applied Medical Sciences, Northern Border University, Arar, Kingdom of Saudi Arabia; Abasyn University, Peshawar, Pakistan, PAKISTAN

## Abstract

A major obstacle in treating malaria is the emergence of antimalarial drug resistance. The aminoacyl-tRNA synthetases (aaRSs), which are essential enzymes for protein synthesis, have been identified as potential targets for the discovery of anti-parasitic drugs. Of these, Glutamyl-tRNA synthetase (GluRS) is a crucial aaRS enzyme in *Plasmodium falciparum* and is a vital therapeutic target. In this study, potential GluRS inhibitors are reported from the Medicinal Fungi Secondary Metabolites and Therapeutics (MeFSAT) chemical library using structure based virtual screening via PyRx v0.8. To evaluate the stability of top hit protein-inhibitor complexes, molecular dynamics simulations (time period, 100 ns) were performed from an initial batch of 1,830 compounds. The two shortlisted compounds, MSID000152 and MSID000974, showed the strongest negative binding affinities of −10.4 kcal/mol and −10.1 kcal/mol, respectively. For comparative analysis, Chloroquine was used as a control to validate the screening results. The dynamics of both complexes showed stable structure, with mean RMSD of 3.94 Å for MSID000152, 2.35 Å for MSID000974, and 2.39 Å for chloroquine. The MSID000152 illustrated favorable MMPB/GBSA binding free energy. Taken together, the study highlights MSID000152 and MSID000974 as promising antimalarial lead compounds for additional in vitro and in vivo investigations.

## Introduction

Roughly one-third of the world’s population suffers from malaria, mostly in South America, Africa, and Asia [1]. Worldwide, there were over 219 million cases of malaria in 2017 and about 435,000 fatalities from the disease [2]. Female Anopheles mosquitoes carry the illness brought on by parasites from the Plasmodium genus [3]. The bloodstream is home to both asexual and sexual phases of the parasite’s life cycle. The asexual stage of malaria causes clinical symptoms, but the sexual stage generates gametocytes that Anopheles mosquitoes use to transmit the infection between hosts. Human malaria may be caused by five different species of Plasmodium, the most common and deadly of which is *Plasmodium falciparum*. These species include *P. vivax, P. malariae, P. falciparum, P. ovale wallikeri,* and *P. ovale curtisi* [3]. Malaria symptoms are vague and might include fever, headache, chills, vomiting, nausea, diarrhea, muscular pains, and exhaustion. Malaria can occasionally result in consequences such as anemia, jaundice, organ failure, coma, and even death. As opposed to 245 million cases and 568,000 fatalities in 2020, the World Malaria Report of 2022 showed a spike in instances, with 247 million cases and 619,000 deaths in 2021 [3,4].

Currently, five different artemisinin-based combination therapies (ACTs) are employed as the primary treatment for malaria. Sesquiterpene lactones, derived from artemisinin, exhibit potent inhibitory activity against all stages of the *Plasmodium* parasite life cycle within the bloodstream [3]. Although ACTs have substantially reduced malaria incidence, recent reports of *P. falciparum* resistance to artemisinin in Southeast Asia have raised significant concerns regarding their long-term efficacy. Surveillance data further suggest that resistant strains of *P. falciparum* are spreading rapidly, irrespective of the specific therapeutic regimens used [5]. This growing resistance underscores the urgent need for innovative strategies to identify novel therapeutic targets, optimize treatment modalities, and develop new antimalarial agents [3]. Artemether-lumefantrine (Coartem®), atovaquone-proguanil (MalaroneTM), quinine, mefloquine, artemisinin, chloroquine, and primaquine are some of the recommended antimalarial medications. However, the widespread use of these agents, coupled with additional contributing factors, has led to escalating resistance. Such resistance not only compromises treatment efficacy but also increases the risk of anemia, recurrent parasitemia, and progression to severe or potentially fatal disease [3,5].

There is just one officially recognized malaria vaccine, RTS, S (Mosquirix®, GlaxoSmithKline), and it offers erratic, transient protection; nonetheless, there have been reports of malaria protection from BCG immunization. The main goal of malaria vaccine research has been to produce strong T-cell or antibody responses [2]. Recent studies suggest that certain vaccines, such as the Bacillus Calmette–Guérin (BCG) vaccine, can induce long-lasting modifications in the innate immune system. This phenomenon, often referred to as BCG-induced “trained immunity,” is characterized by epigenetic and functional reprogramming of innate immune cells, leading to the acquisition of non-specific memory-like properties. Despite these advances, the persistent global burden of malaria highlights the urgent need for novel and improved strategies in antimalarial drug development to achieve effective disease control [6].

The enzymes known as aminoacyl-tRNA synthetases, or aaRSs, are vital for the production of proteins. Researchers have looked into several aaRSs as possible targets for anti-parasitic medications during the last 10 years [7]. These enzymes are divided into two groups according to how they interact with tRNA and ATP. Class I aaRSs are characterized by a characteristic structure called the Rossmann fold and certain ATP-binding motifs, whereas class II enzymes have a distinct structure [8]. During protein synthesis, aminoacyl-tRNA synthetases (aaRSs) play a crucial role in ensuring the correct pairing of tRNA molecules with their cognate amino acids. While tRNA is not required for amino acid activation by most aaRSs, notable exceptions include glutamyl-tRNA synthetase (GluRS), glutaminyl-tRNA synthetase (GlnRS), and arginyl-tRNA synthetase (ArgRS), where tRNA binding is essential to induce specific binding modes and stable complex formation [9]. In *Plasmodium falciparum*, two distinct forms of GluRS have been identified, including a cytoplasmic variant. [10].

These enzymes have undergone substantial evolution, particularly in connection to GluRS. The route that produces Gln-tRNAGln in some species uniquely uses GluRS. Because of their significance and pervasiveness, aaRSs are ideal targets for novel anti-infective medications [11]. Recent research has produced several inhibitor compounds that are unique to the malaria parasite aaRSs, which may lead to the discovery of novel drugs. These inhibitors can target several regions of aaRSs, including the tRNA binding region, editing site, ATP pocket, and other functional domains. Anti-malarial drug discovery has benefited immensely from structural investigations employing X-ray crystallography, which have shed light on how medications like halofuginone and cladosporin interact with their targets. Novel drugs will encourage specificity against malaria parasite aaRSs [12,13].

In this study, we aim to identify novel inhibitors of *Plasmodium falciparum* GluRS, a key enzyme in protein biosynthesis, by utilizing the Medicinal Fungi Secondary Metabolites and Therapeutics (MeFSAT) antifungal library. The MeFSAT library was chosen because it comprises a curated collection of secondary metabolites derived from therapeutic fungi, which are widely recognized for their structural diversity and broad spectrum of biological activities. By focusing on natural product scaffolds, MeFSAT enhances the likelihood of identifying unique and selective GluRS inhibitors, in contrast to large, synthetic-oriented databases such as ZINC. Our computational strategy integrates virtual screening using PyRx with molecular dynamics (MD) simulations in Amber22 to predict and evaluate candidate compounds with high affinity and specificity for *P. falciparum* GluRS. This workflow is designed to accelerate the early phases of drug discovery by prioritizing promising molecules for experimental validation. By combining natural product diversity with advanced computational approaches, this study seeks to expand the chemical space for antimalarial therapeutics and contribute to the development of effective new drugs against malaria.

### Computational workflow

**Fig 1:**
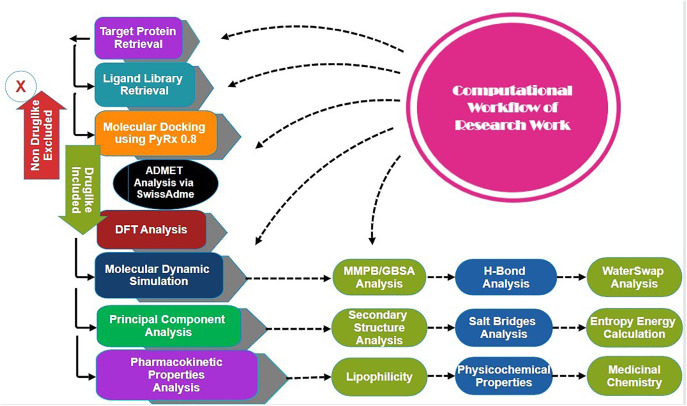
The computational workflow starts with retrieving and preparing the target protein structure, preparing the compound library, and performing molecular docking. This is followed by using ADMET to evaluate the pharmacokinetics profile, conducting DFT analysis to determine electronic properties and structure, and executing MD simulation, MMPBSA, hydrogen Bond analysis, and waterSwap energy estimation. The process further includes principal component analysis (PCA), secondary structure analysis, analysis of salt bridges, and finally, entropy energy calculation.

The schematic workflow of the study is shown in **Fig 1**.

### Target structure retrieval and preparation

The crystal structure of *Plasmodium falciparum* GluRS (PDB ID: 7WAI; 2.1 Å resolution) was used as the target protein, as it provides a high-quality model with a well-defined active site suitable for docking, molecular dynamics (MD) simulations, and subsequent ligand–protein interaction analysis. The structure was initially processed in UCSF Chimera v1.17, where it was examined for residue composition and structural anomalies [14,15]. To relieve steric clashes and optimize geometry, energy minimization was performed using a two-step protocol: 5,000 cycles of the steepest descent algorithm followed by 5,000 cycles of the conjugate gradient method, each with a step size of 0.02 Å [16]. The refined protein structure was then saved in PDB format for use in downstream computational analyses.

### Selection and preparation of ligand library

To identify novel and potent inhibitors of the GluRS enzyme, a dataset of 1,830 natural product-derived compounds was retrieved from the MeFSAT database (https://cb.imsc.res.in/mefsat/download) [17]. The compounds were minimized using the MM2 force field and were subsequently converted into PDBQT format using PyRx v0.8 [1,16,18,19].

### Virtual ligand-protein docking studies

Structure-based virtual screening was performed to evaluate the drug library against the target biomolecule [20]. PyRx v0.8, incorporating AutoDock Vina as the docking engine, was employed to screen the compounds against the target receptor [18,19]. Following the loading of the target protein structure (PDB ID: 7WAI) and the MeFSAT compound library into PyRx, all ligands were subjected to additional energy minimization and converted into AutoDock-compatible format. Docking was then performed, and the top three ligands were selected based on their binding affinity values [17,21]. Compounds exhibiting the lowest binding energies were considered the most stable conformations and were used to rank the ligands [22]. Subsequently, the selected ligands were analyzed, structurally aligned, and visualized in three dimensions (3D) using Discovery Studio v2.4 [23,24].

### SwissADME-based pharmacokinetic evaluation

An essential component of the drug development process is the evaluation of the pharmacological potential and ADME (absorption, distribution, metabolism, and excretion) properties of selected lead compounds [25]. SwissADME tool http://www.swissadme.ch/, was used to assess the compounds’ ADME profile. Furthermore, the pkCSM database at https://biosig.lab.uq.edu.au/ was used to estimate the toxicity of these compounds [23,26].

### Density functional theory (DFT)

**Fig 2:**
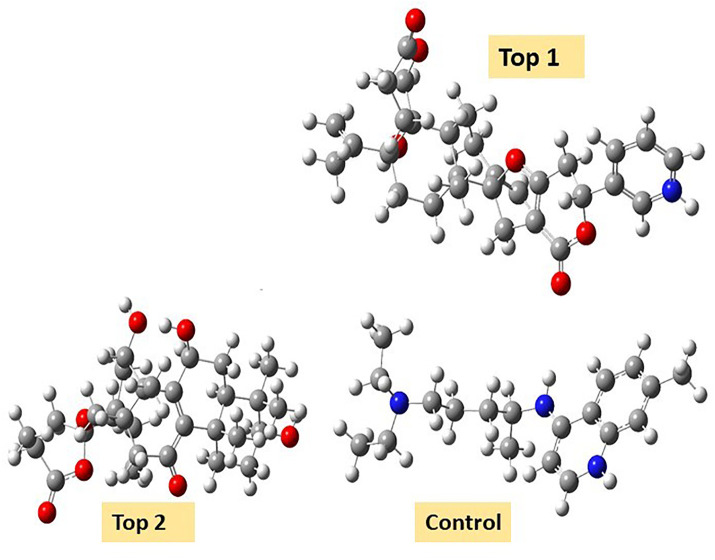
The optimized structures of the investigated compounds.

Density functional theory (DFT) was employed to evaluate the electronic properties and chemical reactivity of the selected compounds [21]. Geometry optimization and frequency calculations were performed using the B3LYP functional with the 6-311G (d, p) basis set [27]. All calculations were conducted with the Gaussian09 software package [28,29]. The Hartree–Fock optimization energy, along with the frontier molecular orbital (FMO) energies, including the highest occupied molecular orbital (HOMO) and the lowest unoccupied molecular orbital (LUMO) and their corresponding energy gap were determined [30,31]. FMO analysis is particularly useful for predicting the stability and reactivity of chemical compounds [32]. The energy gap (Eg = ELUMO – EHOMO) was further used to assess excitation energies and electronic transitions [27]. Additionally, chemical descriptors including chemical potential (μ), hardness (η), softness (σ), electrophilicity index (ω), and electronegativity (χ) were calculated using Koopmans’ theorem to further characterize compound reactivity. Visualization and interpretation of the results were carried out using GaussView06 [33]. **Fig 2** presents the optimized structures of the top-ranked compounds (MSID000152 as **Top 1,** MSID000974 as **Top 2**) alongside the control.

### Molecular dynamic simulations of the docked complexes

Docked complexes were further evaluated using molecular dynamics (MD) simulations, as docking analysis may contain potential inaccuracies [34,35]. Docking primarily identifies the binding site of a ligand within the protein’s active pocket, whereas MD simulations allow for the assessment of ligand–protein interactions under near-physiological conditions, providing insights into binding kinetics and stability [1,36]. All protein–ligand complexes were processed in AMBER22 using the Antechamber module. The receptor protein was parameterized with the FF19SB force field, while ligands were treated using the GAFF2 force field [1,37]. Complexes were solvated in an OPC water box with a 12 Å buffer and heated to 310 K [38]. A production MD simulation was then performed for 100 ns. Structural analyses were conducted using CPPTRAJ, and trajectory plots were generated with XMGRACE v5.1 [1,39,40].

### Analysis of hydrogen bonding patterns

Hydrogen bonding is a critical determinant of ligand–protein binding strength and stability. MD simulation trajectories were analyzed to evaluate hydrogen bond dynamics within the enzyme active site [41]. System preparation, equilibration, and trajectory generation were performed using AMBER22, and hydrogen bond analysis was conducted with the CPPTRAJ module. Hydrogen bonds were identified and quantified using geometric criteria of ≤3.5 Å donor–acceptor distance and ≥120° donor–hydrogen–acceptor angle [42]. Key interactions were further visualized and interpreted using Visual Molecular Dynamics (VMD) software [43,44].

### Estimating binding affinities

Deciphering the interactions between ligands and proteins requires an understanding of the binding free energy [45]. AMBER22 MM/PBSA methods were used to determine the complexes’ binding free energies (G binding). In particular, the binding free energy between the chemicals and the main collagenase enzyme was estimated using MMPBSA [46]. The following formula was used to find the binding energies:


ΔGbind= ΔGsol−TΔS

In Equation 1, ΔGbind represents the total binding free energy, ∆EMM represents the total molecular mechanics energy change, ∆Gsol is the total solvation energy, T represents temperature in Kelvin, and ΔS represents entropy energy upon binding. The product TΔS represents the temperature-dependent entropy contribution to the binding free energy.

### Calculation of entropy energies and waterswap analysis

Entropy energies for each complex were calculated using AMBER’s normal mode estimation, where ten representative frames from the MD trajectories were selected to ensure adequate conformational sampling, and normal mode analysis was performed to estimate entropy contributions [47–49]. To further validate intermolecular complex formation, absolute binding free energies were determined using the advanced WaterSwap approach, which accounts for the critical role of water molecules in mediating ligand–enzyme interactions and provides greater accuracy and efficiency compared to traditional MMPB/GBSA methods [50–52].

### Secondary structure analysis

The proteins of interest underwent secondary structure analysis to identify variations in their secondary structure patterns [53]. The secondary structure of the selected enzyme was analyzed using the AMBER22 VMD module [44].

### Principal component analysis

PCA, also known as Essential Dynamics (ED), is a method for simplifying data that elucidates the observed motional changes of proteins during simulation periods [54]. In this study, PCA was performed by diagonalizing the covariance matrix, which was constructed using the enzyme’s Cα atoms represented in the SMT format, as shown in Equation 2.


C = < (qi−<qi>) (qj−<qj>) T > (2)

Whereas 𝑞𝑖 and 𝑞𝑗 are the Cartesian coordinates of the ith and jth Cα atoms in the targeted enzyme, respectively, and <𝑞𝑖> and <𝑞𝑗> indicate their mean locations over conformational groups generated from MD simulation. Together, the diagonalization-produced eigenvalue and eigenvector represent the structural domain’s coordinated motion and their amplitude fluctuations on an eigenvector, respectively. The PCA was performed using Amber’s CPPTRAJ tool [54]. This approach captures the essential dynamics of the enzyme, providing insights into its structural fluctuations and dynamic behavior throughout the simulation [55].

### Analyzing salt bridges in protein structures

A computational assessment of salt bridges was conducted using the AMBER22 software package [46]. Salt bridges, that develop between amino acid residues in a protein that are oppositely charged, stabilize protein structures and have an impact on molecular interactions [56]. Researchers can identify crucial interactions required for drug binding by using computational analysis of salt bridges to gain a better understanding of the dynamics and stability of protein-ligand complexes [46,57].

### Predictions of pharmacokinetic properties of the complexes

Estimating a compound’s toxicity, excretion, metabolism, distribution, and absorption based on its pharmacokinetics [58]. Using the web server SWISSADME, the pharmacokinetic parameters of the chosen compounds were predicted, and the pkCSM server was used to determine the compounds’ toxicity.

## Results and structural insights

### Retrieval and preparation of GluRS enzyme structure

**Fig 3:**
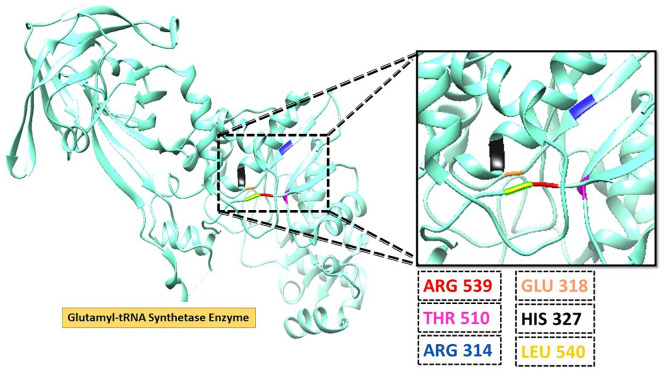
Active site residues in the Glutamyl-tRNA Synthetase enzyme’s three-dimensional structure. The close view of active region is shown and active site residues can be depicted as Arg 539, Thr 510, Arg 314, Glu 318, His 327, and Leu 540.

The present study aimed to identify potential inhibitors of the primary GluRS enzyme in *Plasmodium falciparum*. The crystal structure of GluRS (PDB ID: 7WAI) was retrieved from the Protein Data Bank. The enzyme exhibits a dimeric global stoichiometry (A2) and asymmetric global symmetry (C1). Its structure was resolved at a 2.10 Å resolution using X-ray diffraction. Visualization and structural analysis were performed using UCSF Chimera v1.17. The complete three-dimensional (3D) structure of GluRS, including β-strands, catalytic residues, and α-helices, is illustrated in **Fig 3**. A close-up of the active site, highlighting the residues involved in ligand binding (Arg539, Thr510, Arg314, Glu318, His327, and Leu540), is also presented in **Fig 3**.

### Molecular docking

Molecular docking is a widely used computational technique to predict protein–ligand interactions and plays a crucial role in drug discovery and development [59]. For this study, 1,830 compounds from the MeFSAT library were screened against the *P. falciparum* GluRS enzyme (PDB ID: 7WAI). Virtual screening was performed using PyRx v0.8 with AutoDock Vina. Based on binding scores, ten compounds showing strong interactions with GluRS were shortlisted as potential hits (Table 1). Among these, two compounds (MSID000152 and MSID000974) were selected for further MD simulations to validate docking results and assess their inhibitory potential.

**Table 1:** The docked compounds with the lowest binding energy value (kcal/mol) along with their rank and structure.

Rank	Compound	Library	Structure	Binding Energy
1.	MSID000152	MeFSAT	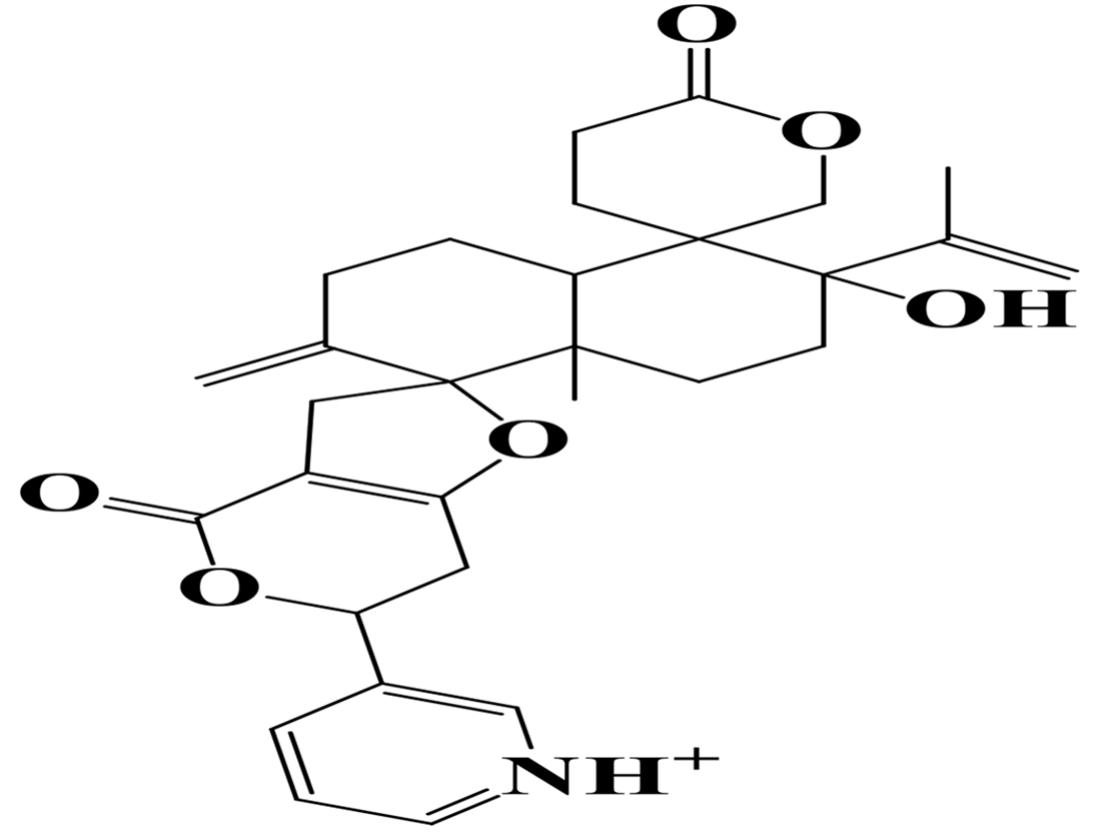	-10.4
2.	MSID000974	MeFSAT	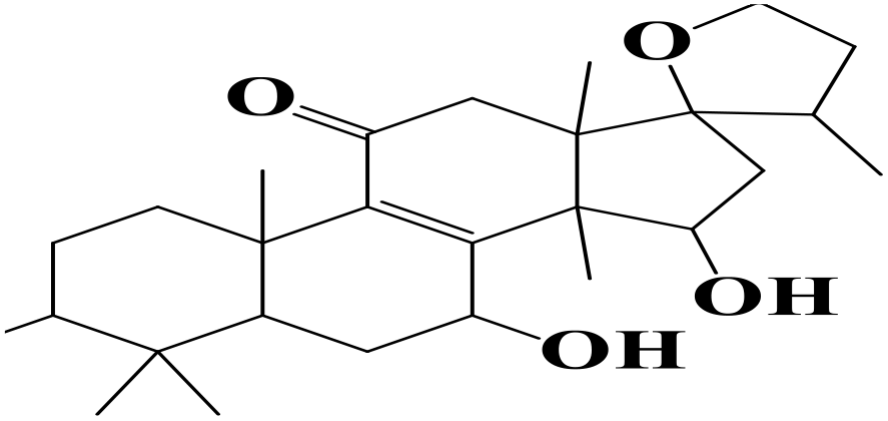	-10.1
3.	MSID000983	MeFSAT	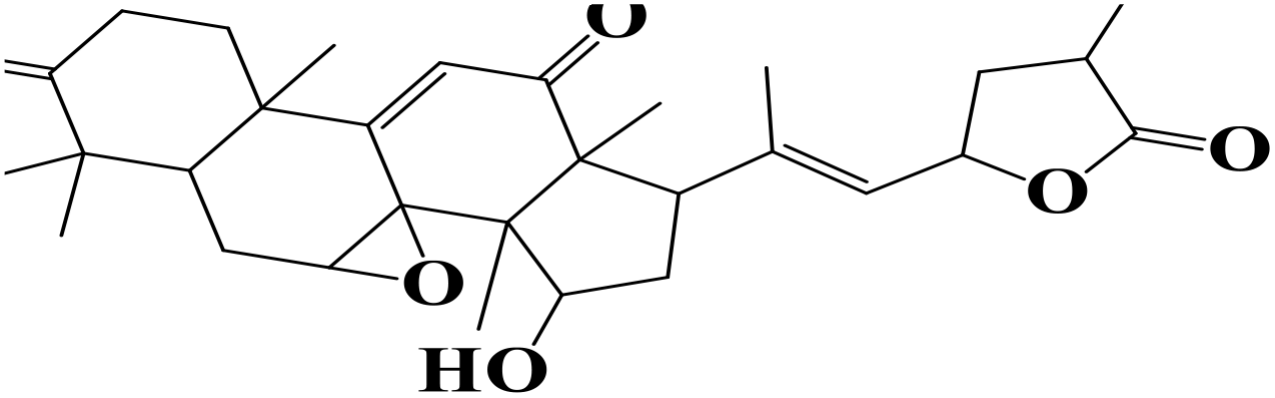	-10.1
4.	MSID000984	MeFSAT	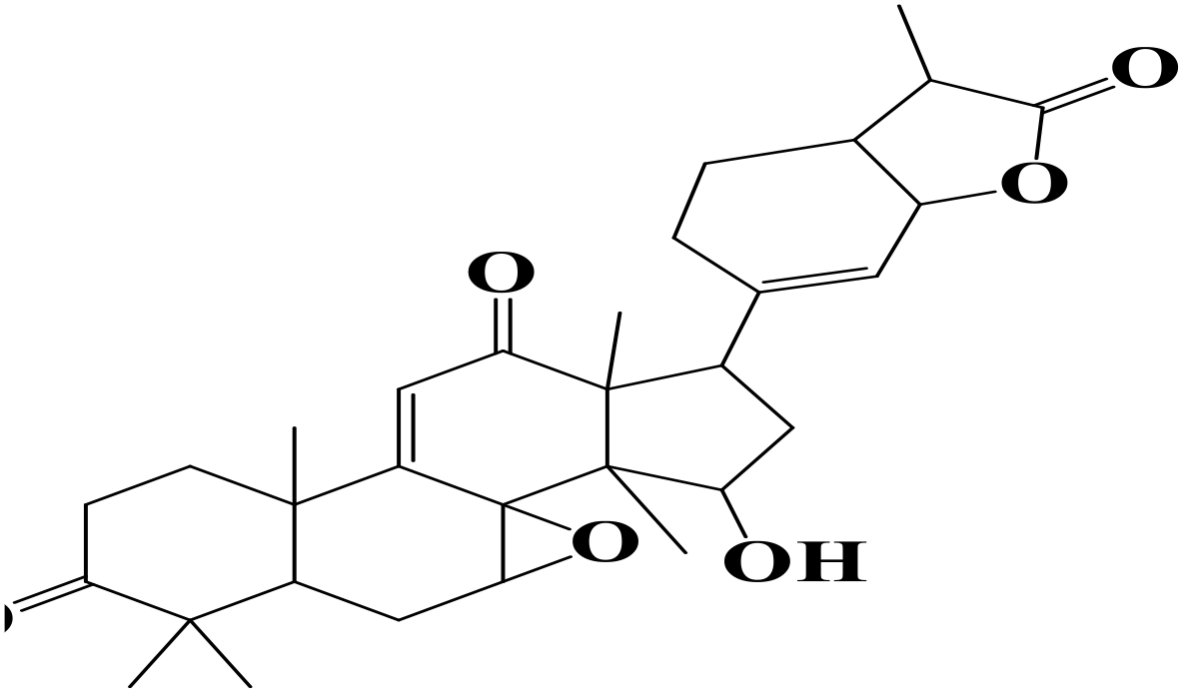	-10
5.	MSID000907	MeFSAT	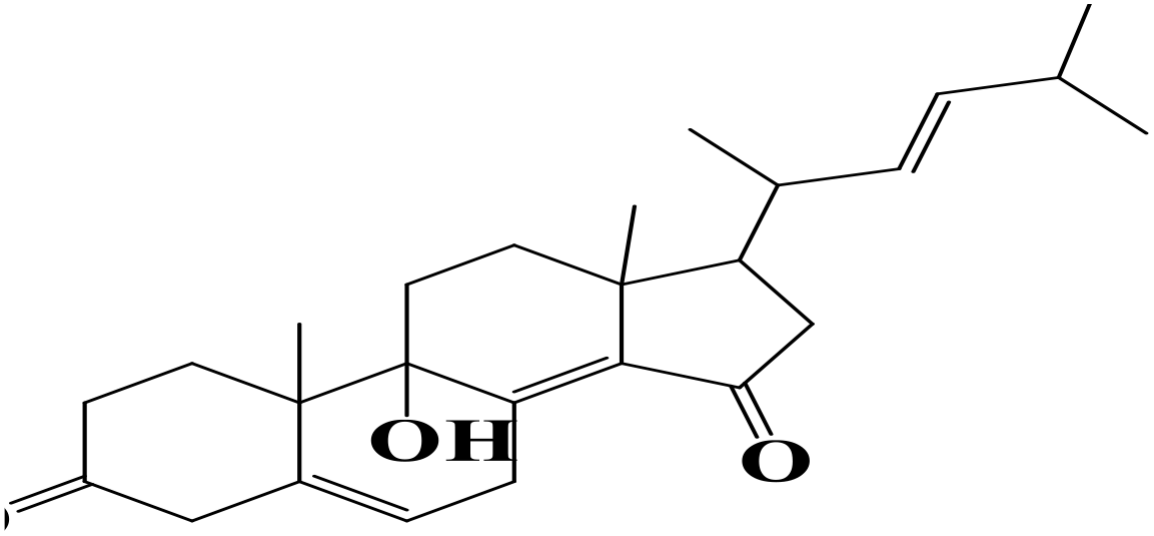	-9.6
6.	MSID000334	MeFSAT	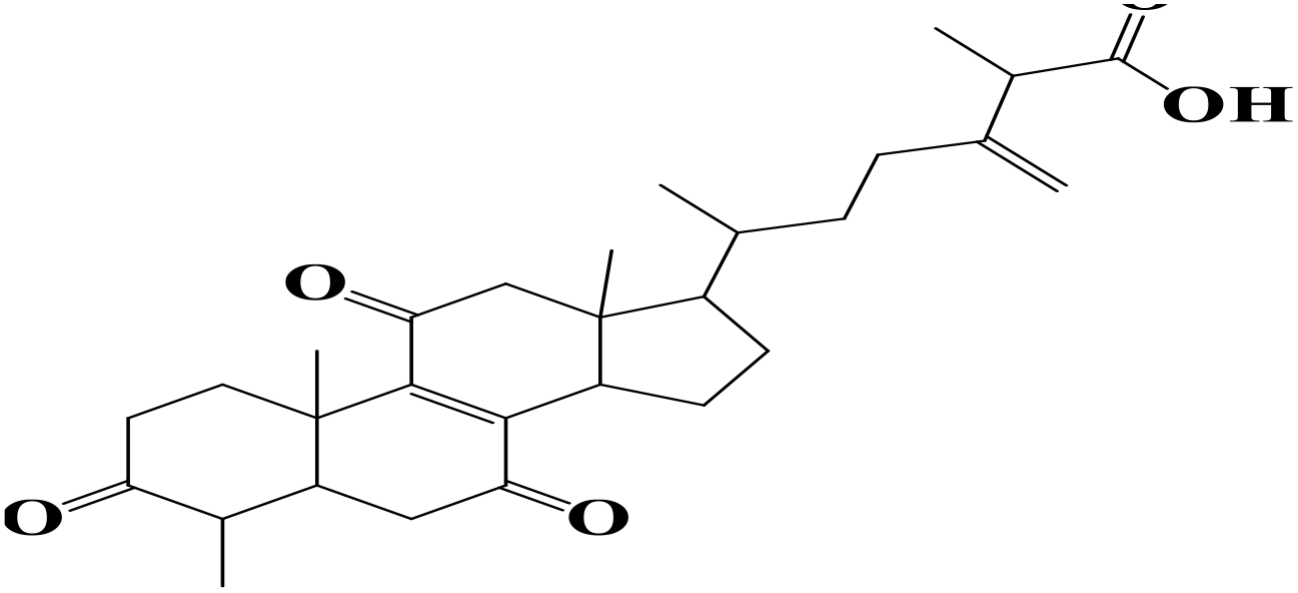	-9.5
7.	MSID000908	MeFSAT	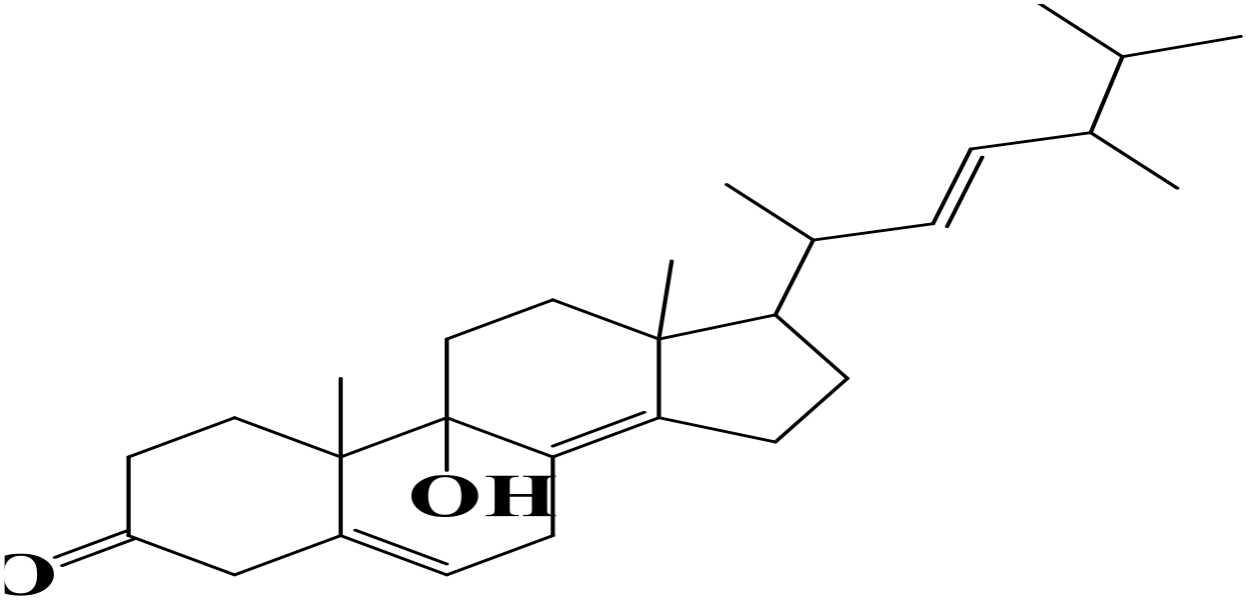	-9.5
8.	MSID000939	MeFSAT	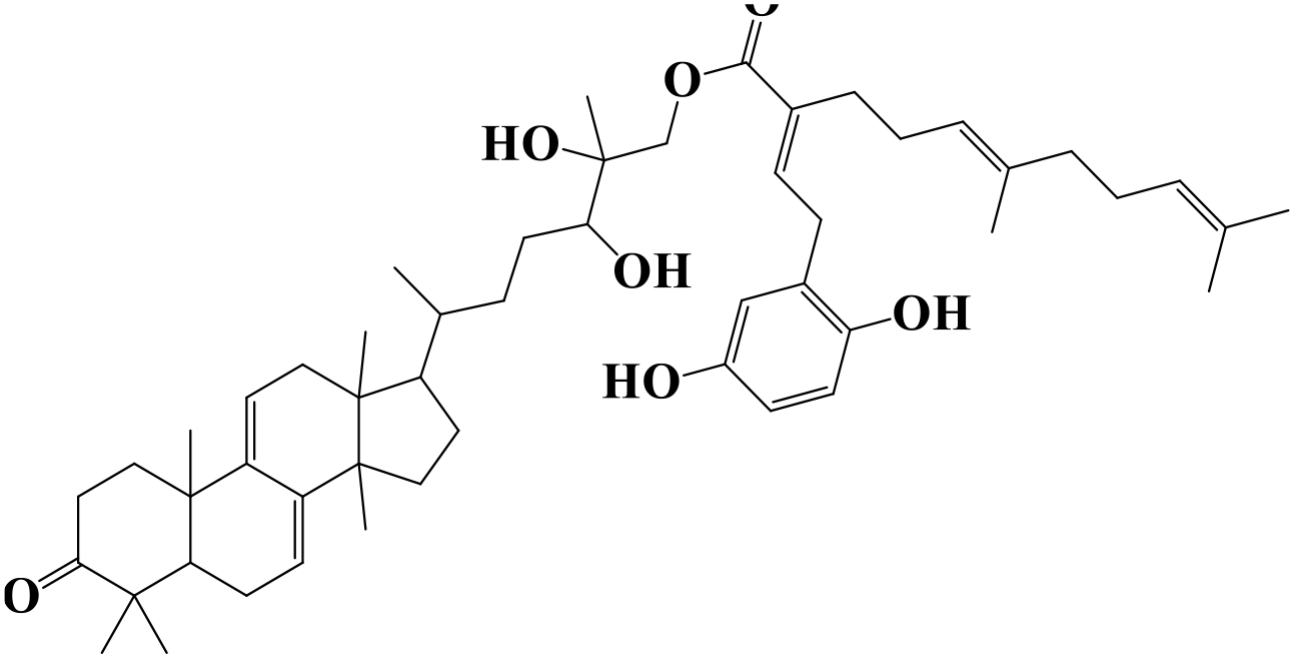	-9.5
9.	MSID000294	MeFSAT	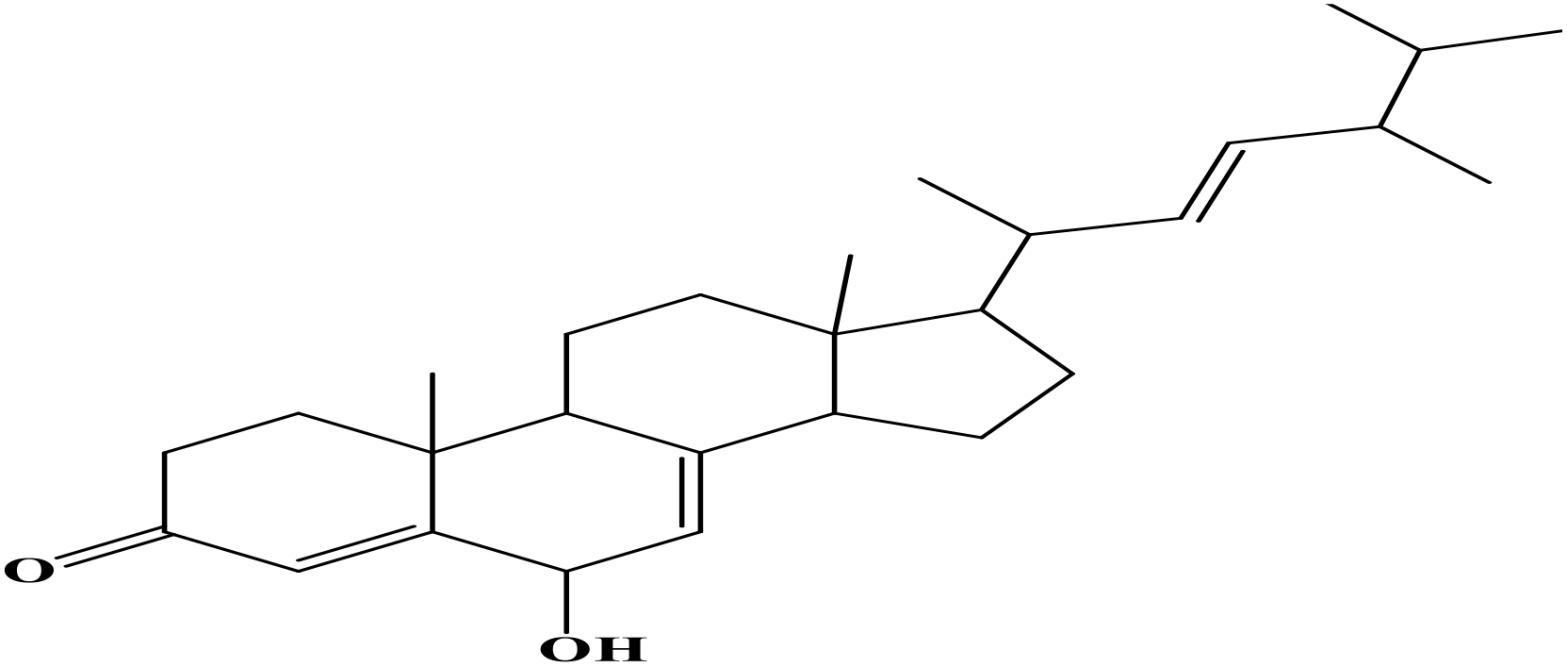	-9.4
10.	MSID000504	MeFSAT	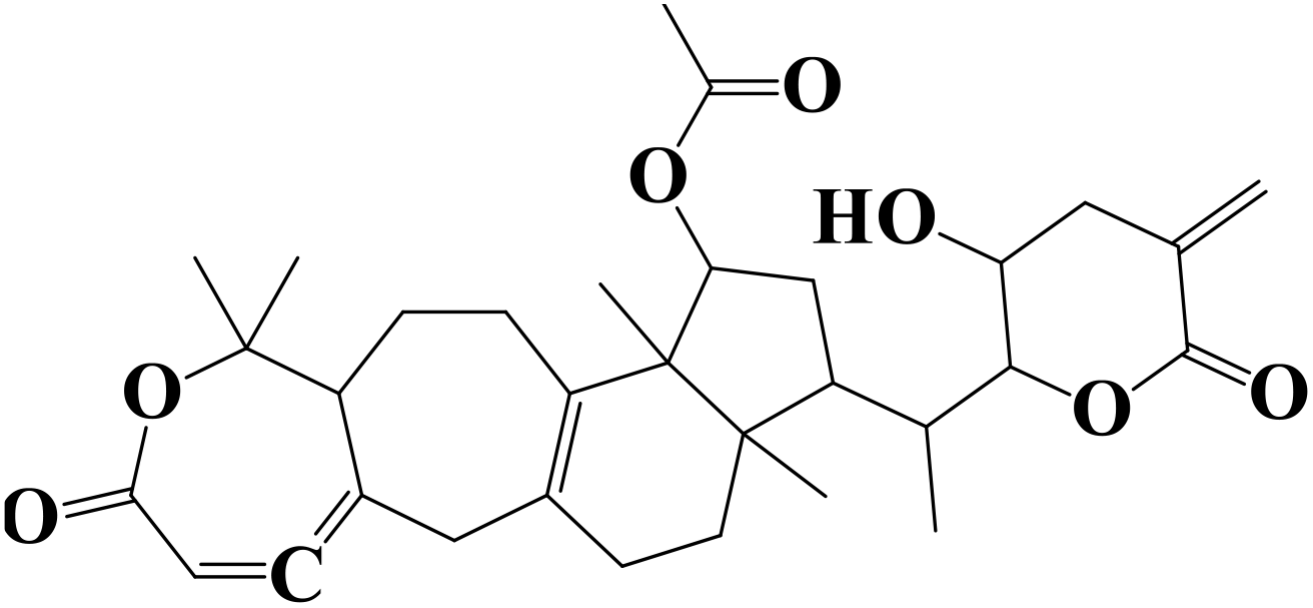	-9.4
11.	Control (Chloroquine)		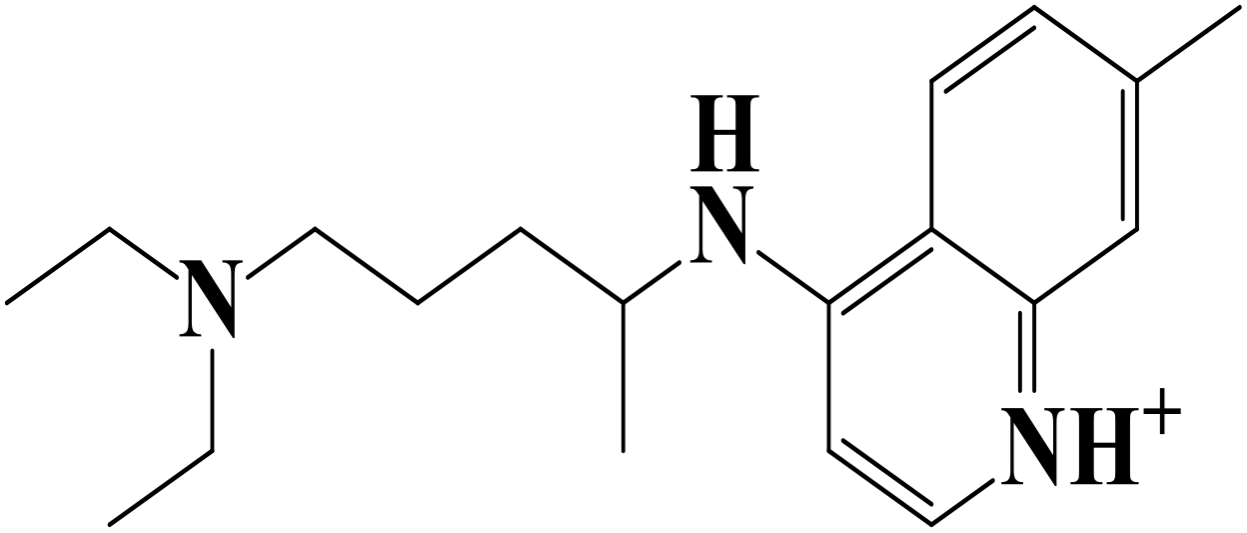	-5.9

**Fig 4:**
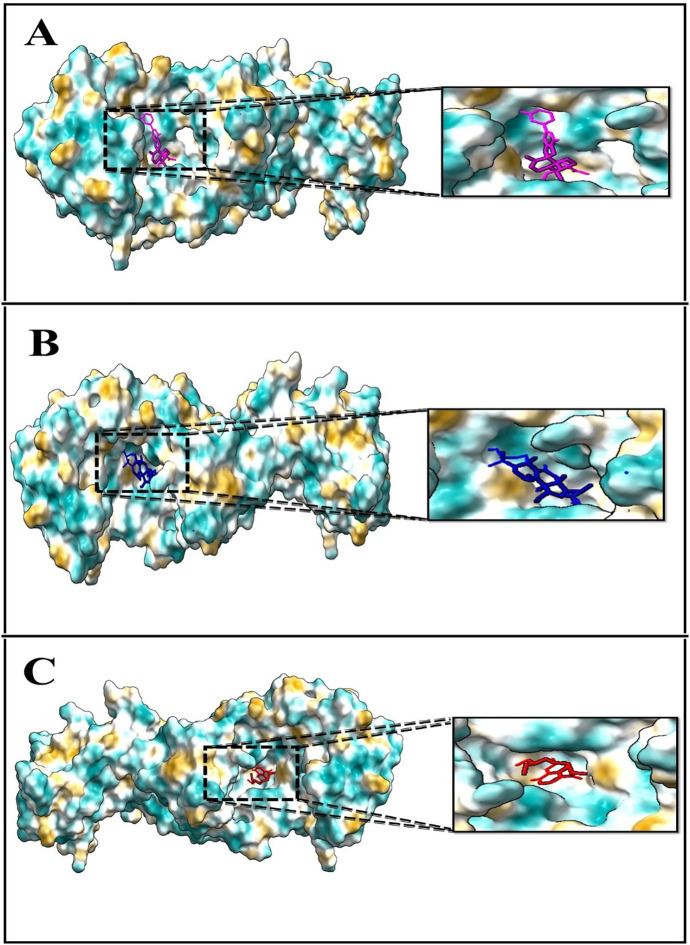
The 3D binding positions and docking interactions of target GluRS with ligands as (A) MSID000152, (B) MSID000974, and (C) Control.

**Fig 5:**
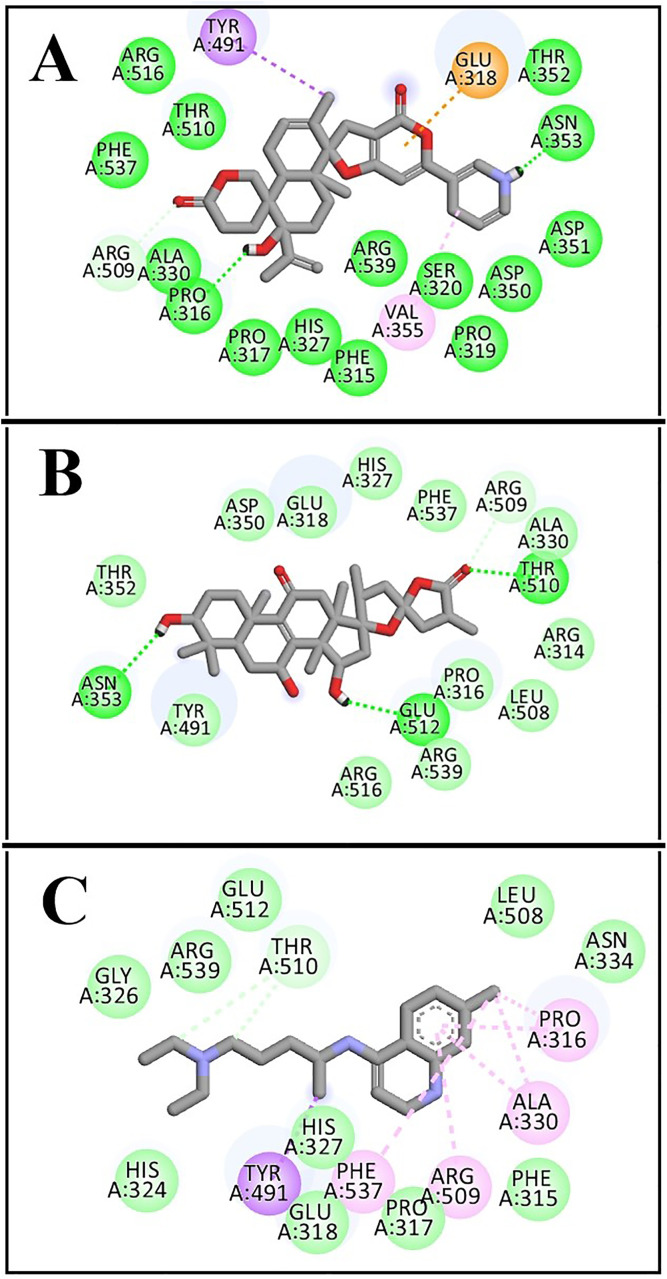
The 2D binding positions and docking interactions of target GluRS with ligands as (A) MSID000152, (B) MSID000974, and (C) Control.

The docking results revealed promising binding affinities, with MSID000152 and MSID000974 showing scores of –10.4 kcal/mol and –10.1 kcal/mol, respectively, compared to –5.9 kcal/mol for the control. **Fig 4 and Fig 5** illustrate the binding modes and interaction regions of the hit compounds. MSID000152, with the strongest binding score (–10.4 kcal/mol), formed conventional hydrogen bonds with SER320, ASN353, and PRO316, along with Pi–Sigma interactions with TYR491, Pi–Anion interactions with GLU318, and Pi–Alkyl interactions with VAL355. MSID000974, the second-best hit (–10.1 kcal/mol), displayed van der Waals interactions with ARG509 and conventional hydrogen bonds with GLU512, ASN353, and THR510, in addition to Pi–Alkyl interactions with PRO316, ALA330, ARG509, and PHE537. The control molecule formed hydrogen bonds only with THR510. **Table 2** summarizes the key interacting residues of GluRS for the top two compounds.

**Table 2:** Amino acids involved in GluRS interactions with compounds.

S.No	Ligand	Interactive Amino Acids
1	**MSID000152**	Arg516, Thr510, Phe537, Tyr491, Arg509, Ala330, Pro316, Pro317, His327, Phe315, Arg539, Val355, Ser320, Asp350, Pro319, Asp315, Asn353, Thr352, Glu318
2	**MSID000974**	Thr352, Asn353, Tyr491, Asp350, Glu318, His327, Phe537, Arg509, Ala330, Thr510, Arg314, Leu508, Pro316, Glu512, Arg539, Arg516
3	**Control**	Glu512, Arg539, Gly326, His324, Tyr491, His327, Glu318, Phe537, Pro317, Arg509, Phe315, Ala330 Pro316, Asn334, Leu508, Thr510

### Density functional theory analysis

**Fig 6:**
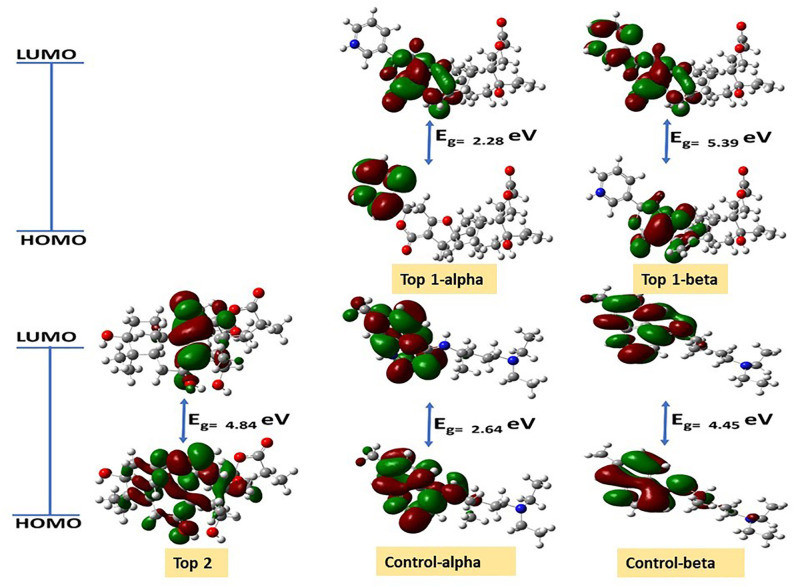
The contour plots of HOMO and LUMO for the investigated compounds MSID000152 as Top 1, MSID000974 as Top 2, and Control.

Table 3 Hartree optimization energy, dipole moment, polarizability, and energy difference of LUMO-HOMO frontier molecular orbitals. The value of the energy gap and dipole moment depicted the charge-transfer propensity of compounds [31]. The compound having a smaller energy gap should be more reactive than one with a large energy gap. The Top-1 was considered more reactive as compared to the other investigated compounds, with the lower value of energy gap (2.28 eV), as shown in **Table 3**. The frontier molecular orbitals with their labeled energy gaps of all compounds are shown in **Fig 6**.

**Table 3:** The optimized energy, dipole moment, polarizability, and energy of HOMO-LUMO orbitals, along with their energy gap.

Ligands	Optimization Energy (a.u.)	Dipole Moment (debye)	Polarizability (a.u.)	E_HOMO_ (eV)	E_LUMO _(eV)	E_g_ (eV)
Top 1-alpha	−1671.06	4.07	366.14	−3.34	−1.05	2.28
Top 1-beta	−1671.06	4.07	366.14	−6.46	−1.07	5.39
Top 2	−1696.48	8.83	350.62	−6.63	−1.80	4.84
Control-alpha	−906.44	2.08	280.07	−2.92	−0.28	2.64
Control-beta	−906.44	2.08	280.07	−5.59	−1.14	4.45

**Fig 7:**
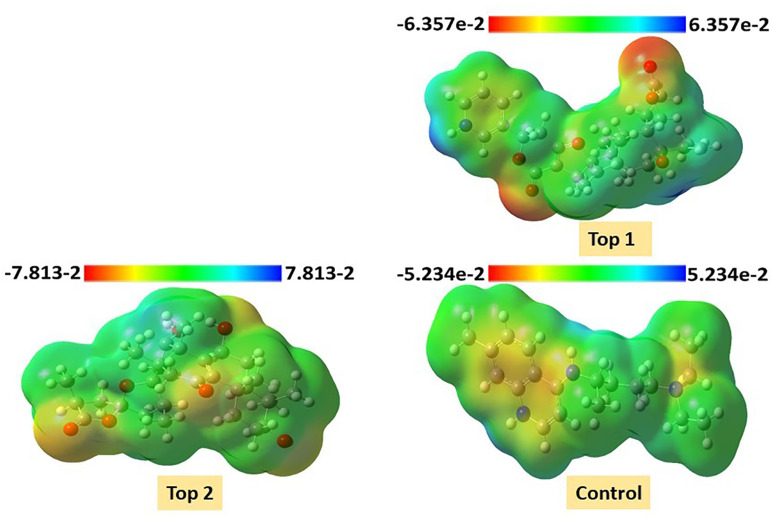
The molecular electrostatic potential (MEP) maps of investigated compounds MSID000152 as Top 1, MSID000974 as Top 2, and Control. The MEP maps were drawn to identify the maximum electropositive and maximum electronegative region within the molecule. The MEP representation showed that the red highlighted region has susceptibility to nucleophilic attack while the blue highlighted region facilitated the electrophilic attack.

Table 4 Shows the values of the global reactivity descriptors, significantly important for exploring the reactivity of the compounds. The values of chemical potential and electronegativity are correlated with each other. The higher value of electronegativity and chemical potential is associated with the ability to attract electrons. The electronegativity values in **Table 4** represent that **Top-2** has a greater electronegativity value than the other compounds. The softness has been associated with reactivity, while the hardness is associated with the stability of the compounds. The **Top-1** compound with a lower hardness value of 0.61 and a higher softness value of 1.80 as compared to all other compounds was identified as a more reactive agent as shown in **Table 4**. The **Top-1** compound behaves like both the good nucleophile and good electrophile with electrophilicity index values of 1.48 and 15.30, respectively. The MEP of each compound is provided in Fig 7.

**Table 4:** Global reactivity descriptors derived from FMO for the compounds.

Ligands	Chemical potential(µ)	Absolute electronegativity (χ)	Chemical hardness (η)	softness(S)	Electrophilicity index (ω)
Top1-alpha	2.19	−2.19	0.61	0.31	1.48
Top1-beta	3.76	−3.76	2.16	1.08	15.30
Top2	4.21	−4.21	1.52	0.76	13.49
Control-alpha	1.60	−1.60	1.18	0.59	1.51
Control-beta	3.36	−3.36	1.66	0.83	9.36

### Molecular dynamic simulation analysis

**Fig 8:**
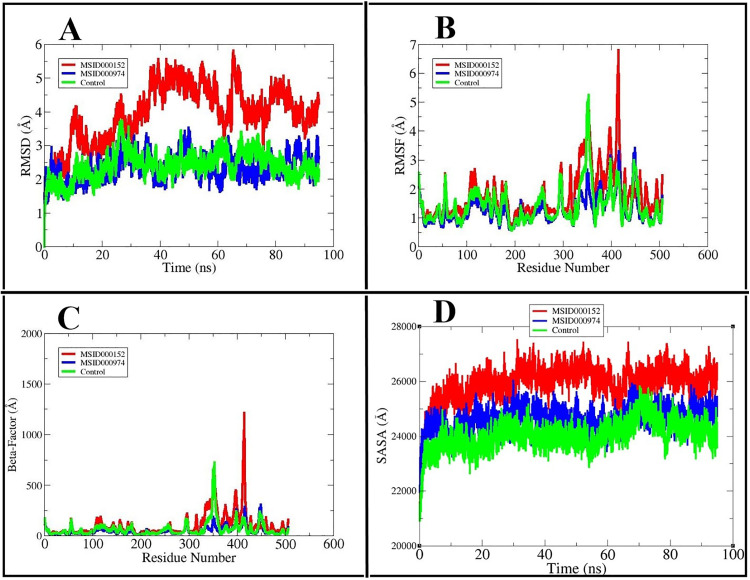
Illustration of flexibility and stability of MSID000152, MSID000974 and the Control molecule using the following analysis: (A) RMSD, (B) RMSF, (C) β-factor, and (D) SASA.

MD simulation was performed on the top docked complexes and the control molecule using AMBER22. Simulation trajectories were analyzed for solvent-accessible surface area (SASA), radius of gyration (RoG), β-factor, and root mean square deviation (RMSD). RMSD plots were used to evaluate the stability of protein–ligand complexes throughout the simulation. A steadily rising RMSD indicates structural deviation, whereas a plateau denotes equilibrium [59]. Both the control molecule and MSID000974 maintained stability without major deviations, while MSID000152 exhibited the highest structural fluctuations. The average RMSD values of the ligand binding poses were 3.94 Å, 2.35 Å, and 2.39 Å, with maximum deviations of 5.81 Å, 3.53 Å, and 3.74 Å, respectively (**Fig 8A**). Root mean square fluctuation (RMSF) analysis revealed the flexibility of amino acid residues, where troughs correspond to rigid regions and peaks to flexible regions [7]. Notable fluctuations were observed mid-simulation, with stabilization at the beginning and end of the 500 ns run. The minimum RMSF values for MSID000152, MSID000974, and the control were 0.65 Å, 0.58 Å, and 0.55 Å, while maximum fluctuations reached 6.79 Å, 3.41 Å, and 5.24 Å, respectively. Their lowest average RMSF values were 1.77 Å, 1.27 Å, and 1.43 Å (**Fig 8B).** β-factor analysis provided additional insight into atom-level flexibility within the complexes [60]. The lowest values for MSID000152, MSID000974, and the control were 11.30 Å, 9.10 Å, and 8.07 Å, while the highest values reached 1214.4 Å, 307.1 Å, and 724.68 Å. Mean β-factor values were 101.9 Å for MSID000152, 14.93 Å for MSID000974, and 64.77 Å for the control (**Fig 8C**). Residues at binding site positions 22–29 displayed enhanced flexibility across all systems.

SASA analysis quantified solvent interactions with the protein surface (**Fig 8D**). Maximum SASA values were 27,545.7 nm² (MSID000152), 26,064.7 nm² (MSID000974), and 25,854.5 nm² (control), while minimum values were 21,051.5 nm², 21,918.2 nm², and 20,876.1 nm², respectively. The corresponding average SASA values were 25,915.1 nm², 24,604.0 nm², and 24,037.1 nm². Notably, MSID000152 exhibited significant SASA variations post-binding, suggesting altered solvent exposure upon complex formation.

### Radius of gyration

**Fig 9:**
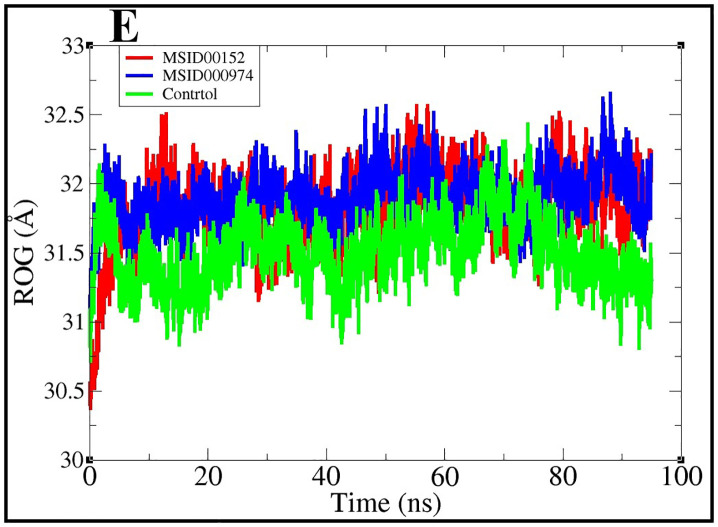
Compactness of MSID000152, MSID000974 complexes, and Control molecules using ROG.

RoG analysis was employed to assess the compactness and structural stability of the protein–ligand complexes throughout the simulation. According to (Akash. *et al*, 2023), an increase in RoG reflects protein expansion or unfolding, whereas a decrease indicates compaction and rigidity. Our results demonstrated that all three complexes (MSID000152, MSID000974, and the control) maintained compact and stable conformations during the 500 ns simulation, supporting the formation of stable protein–ligand assemblies. The average RoG values were 31.82 Å, 31.89 Å, and 31.50 Å for MSID000152, MSID000974, and the control, respectively. The maximum values observed were 32.5 Å, 32.6 Å, and 32.4 Å, while the minimum values were 30.3 Å, 30.8 Å, and 30.7 Å, respectively. Overall, the stable RoG values confirm that ligand binding did not induce significant unfolding, but rather promoted structural compaction and stabilization (**Fig 9).**

### Hydrogen bond analysis of protein-ligand complexes

It was crucial to highlight the significant role of hydrogen bonds in stabilizing the complexes between ligands and docked receptors. Identifying the enzyme residues that formed hydrogen bonds with the compounds was particularly important. As shown in the **Table 5**, the hydrogen atom that forms the bond is referred to as the “Donor,” while the negatively charged atom that accepts the bond is labeled as the “Acceptor.” The “Occupancy” column indicates the percentage of the simulation time during which the hydrogen bond is present.

**Table 5:** Hydrogen bonds formed between ligands and the active site of GluRS. It specifies the donor and acceptor residues involved in the bonding, along with the occupancy percentage, indicating the stability of these hydrogen bonds during the simulation.

No of H-Bonds	Ligand	Donor	Acceptor	Occupancy
Found 3	MSID000152	LIG507-Main	TYR21-Main	31.50%
		HIE26-Side	LIG507-Main	31.60%
		LIG507-Side	GLU17-Side	1.60%
Found 8	MSID000974	LIG507-Side	PRO15-Main	31.60%
		ARG208-Side	LIG507-Main	0.30%
		ARG208-Side	LIG507-Main	0.10%
		LIG507-Side	LIG507-Side	1.00%
		LIG507-Side	THR209-Side	0.40%
		LIG507-Side	ASP49-Side	2.40%
		LIG507-Side	GLU211-Side	31.60%
		LIG507-Side	GLU17-Side	41.00%
Found 4	Control	LIG507-Main	THR-209-Side	69.10%
		LIG507-Side	PRO15-Main	12.60%
		LIG507-Side	HIE26-Side	0.40%
		GLU17-Main	LIG507-Side	0.10%

### Principal component analysis of protein-ligand complexes

**Fig 10:**
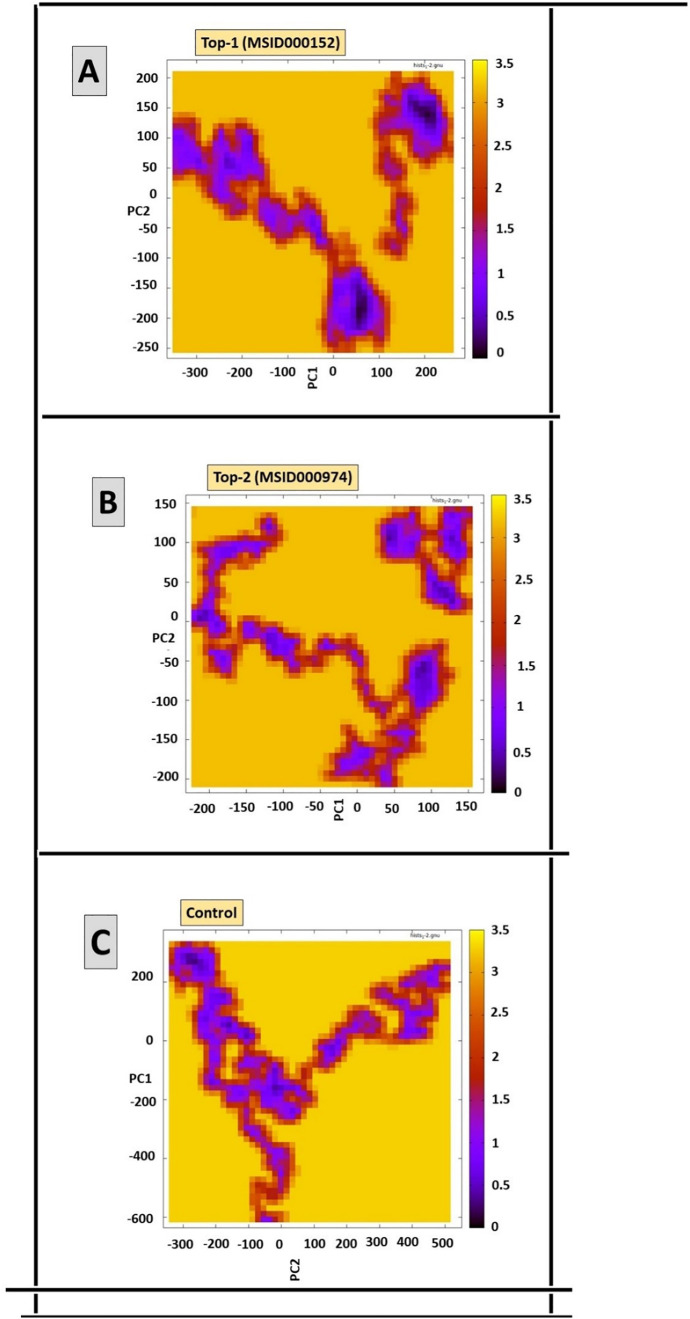
PCA analysis for MSID000152, MSID000974, and Control, respectively.

PCA is one of the multidimensional and polyvariate statistical techniques used to better comprehend the complexity of the large dataset. Principal components (PCs), which stand for unrelated variables, are created by transforming the measured variables [27]. Since each PC is orthogonal to the other, they are complementary to one another. The dataset’s biggest variability is represented by PC1, which is followed by PCs (PC2, PC3, etc.) [61]. To pinpoint the most significant structural alterations that the suggested protein-ligand complexes displayed after interacting with one another, PCA was carried out and visualized [62]. Target enzyme conformation phases and arrangement with ligands and control molecules were analyzed using PCA on 100 ns MD simulation trajectories (**Fig 10**). The bulk of the substances being studied, according to the study’s conclusions, are physiologically active substances that interact with other enzymes and nuclear receptor ligands to block the GluRS enzyme and cause physiological activities.

### Secondary structure analysis of the protein-ligand complexes

**Fig 11:**
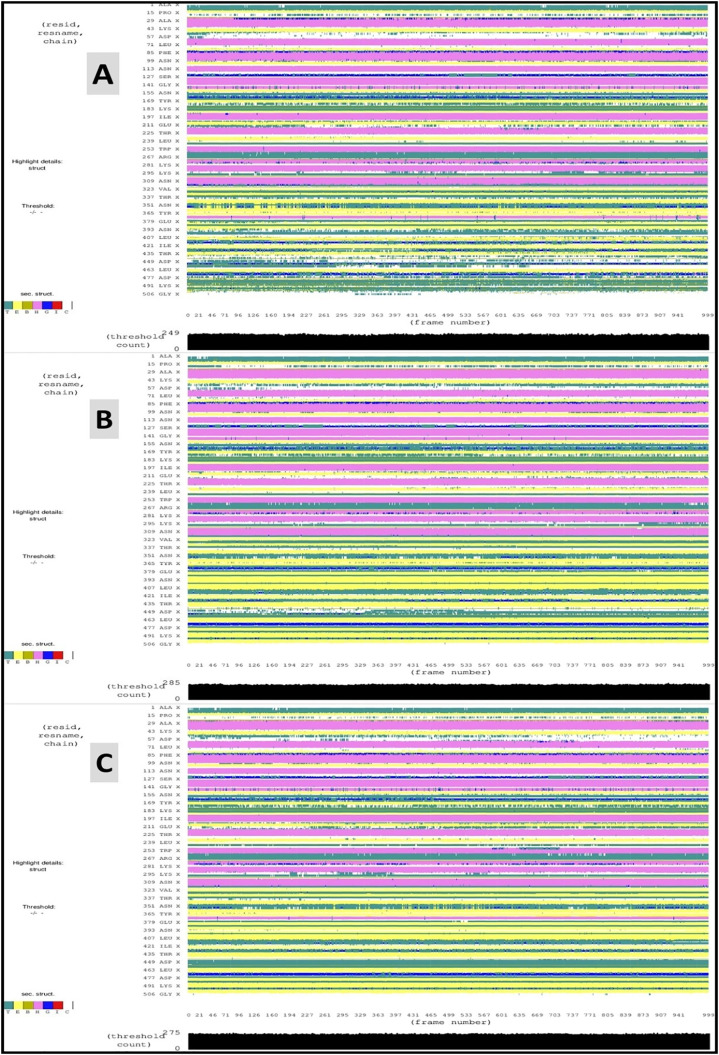
Secondary structure analysis for MSID000152 (A), MSID000974 (B), and Control (C).

The overall architecture of the protein–ligand complexes is stabilized by secondary structural elements, such as α-helices and β-sheets, which also house the active sites essential for enzymatic activity [63]. To investigate the structural underpinnings of *P. falciparum* GluRS function, secondary structure analysis was performed over the course of the simulation. The enzyme displayed tightly packed turns and loops that contribute to substrate binding and catalytic turnover (**Fig 11**). The analysis revealed diverse secondary structural motifs, including: T (turns and loops), E (β-strands), B (isolated hydrogen-bonded β-bridges), H (α-helices), G (3_10_-helices), π (π-helices, a rare helical type), and C (coils). These elements collectively reflect the structural dynamics and adaptability of the enzyme in complex with ligands. Furthermore, **Fig 11** also illustrates the secondary structure distribution for MSID000152 (A), MSID000974 (B), and the control (C).

### MMPB/GBSA analysis

The binding free energies of the MSID000152, MSID000972, and Control docked complexes were computed using AMBER22’s MM-GBSA and MM-PBSA modules. The MM-GBSA computed a total energy of −79.42 kcal/mol for the docked complex MSID000152, −66.52 kcal/mol for the docked complex MSID000972, and −65.52 kcal/mol for the docked complex Control. The computed net free energy for complexes in the MM-PBSA findings was −78.07 kcal/mol, −64.61 kcal/mol, and −64.83 kcal/mol, respectively, according to the data in **Table 6**. Based on the relatively low net binding energy scores, it is expected that the complexes would form robust and stable intermolecular interactions.

**Table 6:** MMPBSA/GBSA binding free energies (in kcal/mol) for MSID000152, MSID000972, and Control docked complexes.

MM/GBSA			
Parameter	MSID000152	MSID000974	Control
Energy Vander Waals	−71.36 kcal/mol	−65.09 kcal/mol	−60.97 kcal/mol
Energy Electrostatic	−22.64 kcal/mol	−18.49 kcal/mol	−15.43 kcal/mol
Total Gas Phase Energy	−94 kcal/mol	−83.58 kcal/mol	−76.4 kcal/mol
Total Solvation Energy	14.58 kcal/mol	17.06 kcal/mol	10.88 kcal/mol
Net Energy	−79.42 kcal/mol	−66.52 kcal/mol	−65.52 kcal/mol
**MM/PBSA**			
Energy Vander Waals	−71.36 kcal/mol	−65.09 kcal/mol	−60.97 kcal/mol
Energy Electrostatic	−22.64 kcal/mol	−18.49 kcal/mol	−15.43 kcal/mol
Total Gas Phase Energy	−94 kcal/mol	−83.58 kcal/mol	−76.4 kcal/mol
Total Solvation Energy	15.93 kcal/mol	18.97 kcal/mol	11.57 kcal/mol
Net Energy	−78.07 kcal/mol	−64.61 kcal/mol	−64.83 kcal/mol

### WaterSwap analysis

**Fig 12:**
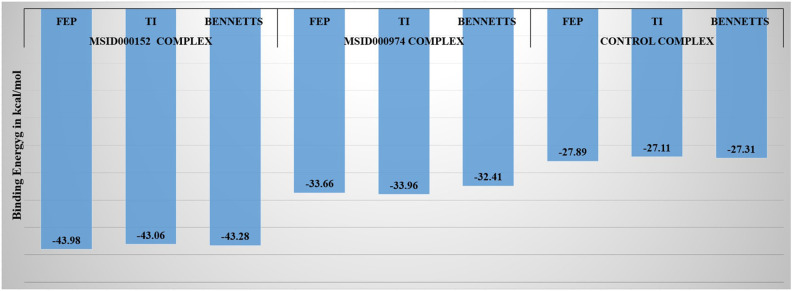
MSID000152, MSID000974, and control molecules’ WaterSwap binding energies are shown in a bar graph. An estimate of the binding energy was calculated and given in kcal/mol.

In computer-aided drug design, water swap energy estimation is used to gain an understanding of how water molecules interact with target proteins and medications [50]. We can create more effective medications by researching these interactions and developing compounds that attach more firmly and precisely to their targets [64]. The binding free energy for every docked complex has been determined using the WaterSwap approach to revalidate the GBSA/MMPBSA calculations. The anticipated WaterSwap binding energy from several phases is shown in **Fig 12**. Specifically, Bennetts, thermodynamic integration (TI) and free energy perturbation (FEP) were the three methods employed. With extremely low binding energies, the lead compound MSID000152 is a good candidate for the drug. **Fig 12** below lists the binding free energy values for each complex for Bennetts, TI, and FEP.

### Entropy energy calculation

In drug development, energy calculations are used to evaluate the stability and binding affinity of ligand-target complexes, providing information on the complex’s structural characteristics, stability, and binding affinity [48]. AMBER 22 was used to compute the entropy energy contribution to get insight into the net binding energy. **Table 7** describes the entropy energy contribution of the top 2 complexes and control, with values for MSID000152, MSID000974, and Control of 4.22, 10.51, and 15.83 kcal/mol, respectively.

**Table 7:** Entropy energy calculations for the docked complexes MSID000152, MSID000974, and Control.

Complex	Translational	Rotational	Vibrational	ΔS Total
**MSID000152**	8.64	17.69	1201.36	4.22
**MSID000974**	14.55	25.34	1524.74	10.51
**Control**	19.62	28.46	1854.15	15.83

### Salt bridges analysis

**Fig 13:**
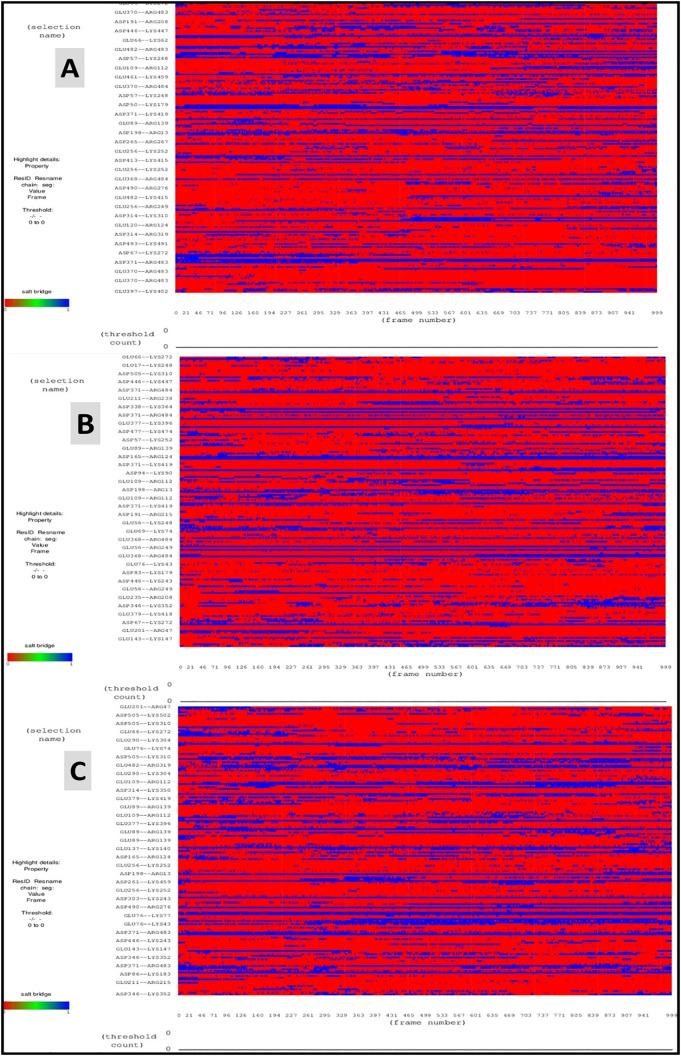
Shows Salt Bridges Analysis for MSID000152 (A), MSID000974 (B), and Control (C).

Salt bridges, which are important in molecular recognition, protein-protein interactions, and protein folding, are the strongest non-covalent interactions discovered in nature [65]. In a salt bridge interaction, two different kinds of side chains take part: Glu or Glu if the ligand has a positive charge, and Arg or Lys if the amino acid chain has a negative charge [66]. This method showed that the docking regions of MSID000152, MSID000974, and Control had electrostatic contact across ions with opposing charges (**Fig 13**). The detailed salt bridges interactions of MSID000152, MSID000974, and Control are given in Table 8.

**Table 8:** Salt Bridges interactions of the ligands MSID000152, MSID000974, and Control with the target protein GluRS.

Complexes	Salt Bridges Interaction
MSID000152	Asp446-Lys447, Glu-Lys62, Glu482-Arg483, Asp57-Lys248, Glu109-Arg112, Glu461-Lys459, Glu370-Arg484, Asp57-Lys248, Asp50-Lys179, Asp371-Lys419, Glu89-Arg139, Asp198-Arg13, Asp265-Arg267, Glu256-Lys252, Asp413-Lys415, Glu256-Lys252, Glu368-Arg484, Asp490-Arg276
MSID000974	Glu66-Lys272, Glu17-Lys248, Asp505-Lys310, Asp446-Lys447, Asp331-Arg484, Glu211-Arg238, Asp338-Lys364, Asp371-Arg484, Glu337-Lys396, Asp477-Lys474, Asp57-Lys252, Glu89-Arg139, Asp165-Arg124, Aps371-Lys419, Asp94-Lys90, Glu109-Arg112, Asp198-Arg13, Glu109-Arg112, Asp371-Lys419, Asp191-Arg215, Glu56-Lys248, Glu69-Lys74, Glu368-Arg484, Glu56-Arg249, Glu368-Arg484, Gly76-Lys43, Asp83-Lys179, Asp446-Lys243, Glu56-Arg249, Glu235-Arg208, Asp346-Lys352, Glu379-Lys418, Asp67-Lys272, Glu201-Arg47, Glu143-Lys147
Control	Glu201-Arg47, Asp505-Lys502, Asp505-Lys310, Glu66-Lys272, Glu290-Lys304, Glu76-Lys74, Asp505-Lys310, Glu482-Arg319, Glu290-Lys304, Glu109-Arg112, Asp314-Lys350, Glu379-Ly419, Glu89-Arg139, Glu109-Arg112, Glu377-Lys396, Glu89-Arg139, Glu89-Arg139, Glu137-Lys140, Asp165-Arg124, Glu256-Lys252, Asp165-Arg124, Glu256-Lys252, Asp198-Arg13, Asp261-Lys459, Glu256-Lys252, Asp303-Lys243, Asp490-Arg276, Glu76-Lys77, Glu76-Lys43, Asp371-Arg483, Asp446-Lys242, Glu143-Lys147, Asp346-Lys352, Asp371-Arg483, Asp86-Lys183, Glu211, Arg215, Asp346-Lys352

### Drug-likeness properties prediction

Determining a molecule’s likelihood of commercialization requires predicting its drug-likeness. Drug-like substances usually have good oral absorption and bioavailability, which guarantees efficient distribution to target locations [67]. Strong drug-like qualities were shown by the analysis, which showed that the lead compounds and control followed the Lipinski rule of five [68]. It appears that the tested compounds attach to biomolecules selectively since none of them triggered PAINS alarms [69]. Acceptable synthetic accessibility ratings also suggest that these compounds are readily synthesized for medicinal chemistry applications, offering a strong basis for additional investigation and advancement [70]. **Table 9** provides an overview of the compounds’ lead profiles, drug similarity, and medicinal chemistry properties. This table offers insightful information about their potential for more research and use in drug discovery initiatives.

**Table 9:** Estimated medicinal chemistry properties and drug-like properties of specific lead compounds and the control molecule.

Complex	Lipinski	Ghose	Veber	Egan	Muegge	Bioavailability Score	PAINS	Synthetic Accessibility
MSID000152	Yes; 1 violation MW > 500	No; 3 violations MW > 480; MR130, #atoms>70	Yes	Yes	Yes	0.55	0 alert	6.64
MSID000974	Yes; 1 violation MW > 500	No; 3 violations MW > 480; MR130, #atoms>70	Yes	Yes	Yes	0.55	0 alert	7.45
Control	Yes, 0 violation	Yes	Yes	Yes	Yes	0.55	0 alert	2.70

### Pharmacokinetic properties of the compounds

Based on predictions about the compounds’ physicochemical properties, lipophilicity, and pharmacokinetic properties, attractive profiles for therapeutic development are indicated. The compounds’ molecular weights are within the acceptable range, indicating that the Lipinski rule of five is followed [71]. Good membrane permeability is suggested by their large topological polar surface area (TPSA), and targeted delivery of high dosages is made easier by their superior water solubility and oral administration [72]. Furthermore, the molecules demonstrate effective absorption into the gastrointestinal system. Taken together, these advantageous characteristics—which are listed in Table Table 10–11, and Table 12 support these compounds’ potential as effective therapeutic options.

**Table 10:** pharmacokinetic properties of compounds.

Pharmacokinetic Properties									
Complex	GI Absorption	BBB permeant	P-gp substrate	CYP1A2 inhibitor	CYP2C19 inhibitor	CYP2C9 inhibitor	CYP2D6 inhibitor	CYP3A4 inhibitor	Log Kp
MSID000152	High	No	Yes	No	No	No	No	Yes	−6.92 cm/s
MSID000974	High	No	Yes	No	No	No	No	No	−7.35 cm/s
Control	High	Yes	No	Yes	No	No	Yes	No	−5.03 cm/s

**Table 11:** lLpophilicity properties of compounds.

Lipophilicity Properties						
Complex	Log *P*o/w (iLOGP)	Log *P*o/w (XLOGP3)	Log *P*o/w (WLOGP)	Log *P*o/w (MLOGP)	Log *P*o/w (SILICOS-IT)	Consensus Log *P*o/w
MSID000152	3.51	3.48	3.97	2.83	5.11	3.78
MSID000974	3.37	2.96	3.68	2.84	3.81	3.33
Control	3.93	4.37	3.69	2.94	4.20	3.83

**Table 12:** Physicochemical properties of the compounds..

Physiochemical Properties								
Complex	Molecular Weight	TPSA	H-bond acceptors	H-bond donors	Formula	No. of Heavy atoms	No. of aromatic heavy atoms	Molar Refractivity
MSID000152	506.61g/mol	96.20Å²	6	2	C30H36NO6+	37	6	137.85
MSID000974	516.67g/mol	113.29Å²	7	3	C30H44O7	37	0	138.26
Control	300.46g/mol	29.41Å²	1	2	C19H30N3+	22	10	98.26

## Discussion

Research efforts aimed at understanding the molecular basis of disease at the structural level often require substantial time and resources. Experimental approaches, while essential, are frequently limited by technical constraints, cost, and scalability. Consequently, there is a growing reliance on *in silico* strategies, which offer more efficient and precise means of identifying potential inhibitors [16]. These computational approaches integrate both sequence-based and structure-based prediction methods, employing diverse algorithms that collectively enhance the accuracy and reliability of predictions [73].

Malaria continues to be a major global health burden, particularly in regions of South America, Africa, and Asia. It is estimated that nearly one-third of the world’s population remains at risk of infection. In 2017 alone, over 219 million cases of malaria were reported worldwide, resulting in approximately 435,000 deaths [2]. Human malaria is caused by five distinct Plasmodium species, *P. vivax, P. malariae, P. falciparum, P. ovale wallikeri,* and *P. ovale curtisi* with *P. falciparum* being the most prevalent and lethal species [3].

Currently, five different ACTs represent the frontline treatment for malaria. Sesquiterpene lactones, derived from artemisinin, are highly effective as they inhibit multiple stages of the *Plasmodium* parasite life cycle within the bloodstream [3]. Despite their success in significantly reducing malaria incidence, growing concerns have emerged regarding the efficacy of these therapies due to the emergence of *P. falciparum* strains resistant to artemisinin, particularly in Southeast Asia. Surveillance data further indicate that resistant variants of *P. falciparum* are spreading rapidly, regardless of the therapeutic regimen employed [5]. This persistent threat underscores the urgent need for innovative strategies aimed at identifying novel therapeutic targets, exploring alternative treatment approaches, and developing new antimalarial drugs [3].

Protein like aaRSs play an indispensable role in protein synthesis, positioning them as attractive targets for antiparasitic drug development over the past decade [7]. These enzymes are broadly classified into two groups based on their structural features and mode of interaction with tRNA and ATP: Class I aaRSs, which are characterized by a Rossmann fold and conserved ATP-binding motifs, and Class II aaRSs, which adopt a distinct structural configuration [8]. Their primary function is to ensure the accurate pairing of tRNAs with their respective amino acids, a critical step in maintaining translational fidelity. While most aaRSs can catalyze amino acid activation independently of tRNA, notable exceptions include GluRS, GlnRS, and ArgRS, where tRNA binding is essential to induce specific conformational states and complex formation [9]. In *Plasmodium falciparum*, two isoforms of GluRS have been identified, including a cytoplasmic variant, further highlighting its biological importance and potential as a therapeutic target [10].

A thorough in-silico investigation was carried out using computational methods to find putative inhibitors of *P. falciparum’s* primary GluRS enzyme. We screened the 1830 compounds in the MeFSAT library using PyRx (0.8) for molecular docking, and we found 2 lead compounds. Promising binding scores of −10.4 kcal/mol and −10.1 kcal/mol were demonstrated by MSID000152 and MSID000974, respectively. In contrast, the control had a binding score of −5.9 kcal/mol to the active region of the enzyme. During the simulated period, there was no discernible change in the binding mode or interactions. These substances demonstrated good pharmacokinetic characteristics and fulfilled the requirements to be classified as drugs.

Based on the MD simulation outcomes, MSID000152 demonstrated lower stability compared to the control molecule, whereas MSID000974 exhibited stability comparable to the control. The β-factor and RMSF analyses revealed that MSID000152 was more flexible than both the control and MSID000974, a property that likely contributes to its reduced stability. The higher degree of deviation observed for MSID000152 suggests less reliable binding, while the consistent RMSD profiles of MSID000974 and the control indicate sustained ligand binding conformations. SASA analysis further highlighted significant post-ligand binding alterations in MSID000152, which may influence its interaction with solvent molecules and compromise its functional efficiency. In contrast, RoG analysis confirmed that all complexes maintained stable and compact conformations, with MSID000974 and the control remaining slightly more compact than MSID000152. Collectively, these findings suggest that MSID000974, due to its compact structure, stable binding, and overall conformational stability, emerges as the most promising candidate.

According to the H-bond analysis, three hydrogen bonds were produced by MSID000152, with occupancies of 31.50%, 31.60%, and 1.60%; eight hydrogen bonds were made by MSID000974, with occupancies of 31.60% and 41.00% of note. The 100 ns molecular dynamics simulation revealed notable structural alterations according to PCA, indicating that both ligands are physiologically active and may block the enzyme. The existence of crucial structural components, such as alpha helices and beta sheets, was revealed by secondary structure analysis. These structural factors support the stability and functioning of protein-ligand complexes, enhancing their potential for efficient binding and therapeutic use.

Moreover, all three complexes, MSID000152, MSID000974, and Control, formed substantial electrostatic interactions with the GluRS protein, which is essential for stability and binding affinity, according to the salt bridges study. While MSID000974 established a large network of contacts, indicating good stability inside the binding pocket, MSID000152 displayed multiple important salt bridges. Notable salt bridge forms were also seen by the Control, indicating that it interacts with the target protein effectively. All things considered, these interactions highlight how crucial salt bridges are for maintaining ligand-receptor complex stability and may help explain their physiological significance.

Our findings align with previous studies investigating inhibitors of *P. falciparum* aaRSs. For instance, [74] highlighted that parasitic diseases significantly contribute to human morbidity and mortality, with the emergence of drug-resistant strains posing major challenges to effective treatment and eradication. The development of small-molecule inhibitors has reinforced the potential of aaRSs as promising therapeutic targets, given their essential role in protein synthesis. Progress in characterizing aaRSs across a range of parasites including *Trypanosoma*, *Giardia*, *Leishmania*, *Toxoplasma*, *Brugia*, and *Plasmodium*, has provided a solid foundation for further exploration of these enzymes. Such insights not only strengthen the rationale for targeting aaRSs but also open avenues for cross-species application of inhibitors, thereby accelerating the discovery and development of novel anti-parasitic drugs.

Identifying potential *P. falciparum* GluRS inhibitors represents a promising step in addressing malaria, a persistent global health threat. Effective inhibitors could contribute to overcoming drug resistance and improving treatment outcomes, ultimately supporting malaria control and eradication efforts. In this study, computational approaches, including virtual screening, molecular docking, and molecular dynamics simulations provided valuable insights into the inhibitory potential of selected compounds against GluRS. However, it is important to note that these predictions remain theoretical and must be substantiated by experimental validation. The identified hits therefore serve as promising starting points for further research. Future studies should include in vitro enzyme inhibition assays, followed by cellular and *in vivo* antimalarial evaluations, to confirm the biological activity and therapeutic potential of these compounds.

## Conclusion

This study reported MSID000152 and MSID000974 as promising lead compounds against *P. falciparum* GluRS. Both compounds showed stable binding affinity and dynamics with the GluRS enzyme and achieved robust binding interactions. As anti-malarial drug resistance is emerging, the shortlisted leads in this study may provide a parent scaffold for the design of more effective inhibitors, subject to experimental in vitro and in vivo analyses.

## Supporting information

S1 File Highlights. (DOCX)
