# Supplementary material for: Rational design of novel Plasmodium falciparum glutamyl-tRNA synthetase inhibitors for the development of next-generation antimalarial drugs
Source: PLoS One. 2025 Dec 4;20(12):e0334429. doi: 10.1371/journal.pone.0334429 (PMC12677556; doi:10.1371/journal.pone.0334429)
Supplement: S1 File — (DOCX) [file pone.0334429.s001.docx]

- Glutamyl -tRNA Synthetase (PDB ID;7WAI) is a crucial aaRS enzyme in Plasmodium falciparum (Pf) and a crucial target for the treatment of malaria.
- MSID000152 and MSID000974 were the top two compounds found with binding energy scores of -10.4 kcal/mol and -10.1 kcal/mol, respectively.
- MSID000974 stayed exceptionally stable during the simulation.
